# Off the Shelf Cloud Robotics for the Smart Home: Empowering a Wireless Robot through Cloud Computing

**DOI:** 10.3390/s17030525

**Published:** 2017-03-06

**Authors:** Javier Ramírez De La Pinta, José María Maestre Torreblanca, Isabel Jurado, Sergio Reyes De Cozar

**Affiliations:** 1 Departamento de Ingeniería de Sistemas y Automática, Escuela Superior de Ingenieros, Universidad de Sevilla. Camino de los descubrimientos s/n, 41092 Sevilla, Spain; jarapi@gmail.com (J.R.D.L.P.); sergio.reyesdecozar@gmail.com (S.R.D.C.); 2 Departamento de Ingeniería, Universidad Loyola Andalucía, Campus de Palmas Altas 41014, Spain; ijurado@uloyola.es

**Keywords:** smart home, service robots, cloud computing

## Abstract

In this paper, we explore the possibilities offered by the integration of home automation systems and service robots. In particular, we examine how advanced computationally expensive services can be provided by using a cloud computing approach to overcome the limitations of the hardware available at the user’s home. To this end, we integrate two wireless low-cost, off-the-shelf systems in this work, namely, the service robot Rovio and the home automation system Z-wave. Cloud computing is used to enhance the capabilities of these systems so that advanced sensing and interaction services based on image processing and voice recognition can be offered.

## 1. Introduction

Nowadays, service robots are gradually being introduced into homes. For example, autonomous vacuum cleaners such as Roomba or Navibot have become common devices. Other popular robots are Rovio, which provides users with telepresence services, and the quadrotor ARDrone, which has become a successful domestic recreational device. All of them have contributed to giving a more realistic view of the current capabilities of service robots, still very far away from the expectations generated by science-fiction and futuristic movies.

Besides the proliferation of service robots at homes, other household technologies are being adopted to improve people’s lives and to optimize energy consumption. This is the case of smart home technologies, which provide users with services regarding automation, security, and multimedia management. Unfortunately, the lack of standardization is a major issue that hinders its development [1,2]: many different technologies offer smart home services (e.g., the old X-10, LonWorks, KNX, Z-wave,…), but none of them has become the reference for marketers and developers. For this reason, different middleware platforms have been proposed to integrate heterogeneous technologies in the same network, e.g., OSGi [3], UPnP [4], or Jini [5], to name a few.

In this paper, we deal with the integration of service robots in the smart home. In the literature, different works that deal with this problem have been reported. In References. [6,7], the service robots Roomba and Rovio were integrated into a smart home network using UPnP adapters. A more general approach is provided by DH-Compliant [8,9], which is an architecture tailored for the integration of home automation systems and service robots. Other alternatives are: Miro [10], which is an object-oriented robot middleware that makes the development of mobile robot applications easier and faster, and promotes the portability and maintainability of robot software; ROS (Robot Operating System) [11], which is an open source framework to support modular, tools-based software development; OROCOS (Open Robot Control Software) [12,13,14], which is an open distributed framework for robot control, consisting of object libraries and components, and a standard middleware; and RoboMaidHome [15], which consists of wireless sensor networks for service robots to provide reliable services.

**Figure 1:**
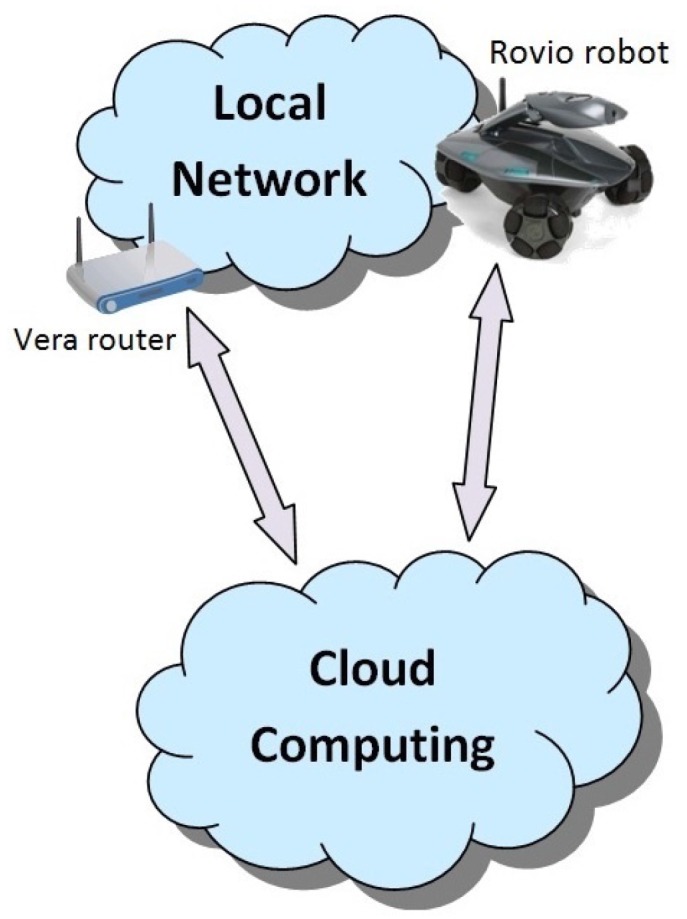
Cloud robotics scenario.

The integration of robots and smart home systems allows for providing new services. However, the development of such services may be limited by the computational power available at home. In particular, the hardware used by robots and smart home systems typically have little computation capabilities. For this reason, we rely here on cloud computing whenever heavy calculations must be performed. We illustrate the approach with a novel case study based on the following off-the-shelf components: Rovio robot and Vera (a low-cost Z-Wave controller). In particular, we have integrated Rovio in a Z-Wave home automation system governed by Vera. The integration of this robot allows us to offer new services based on image processing and voice recognition. Given that these services are computationally expensive, and taking into account that there may not be devices capable of performing the computations in the local network, the solution we propose is to use the cloud. This idea and the whole scenario can be seen in Figure 1.

### 1.1. From Cloud Computing to Cloud Robotics

Cloud computing has become a very popular topic in the last few years, but the concept is not new; many *old* services for e-mailing or file sharing are based on the same principle. The main idea is to provide remote computational resources as services invoked through a network. In this way, it is possible to provide low-resource devices with access to a massive amount of data and computation power, including distributed and parallel processing. In general, the advantages of cloud computing are [16,17]:

- Ease of data exchange with external servers,
- Flexible configuration,
- Middleware: cloud computing servers can provide a virtualized platform for each component in the network to collaborate with each other,
- A server can handle heavy processes so that vendors can manufacture smaller, cheaper and more energy efficient devices,
- High scalability,
- Maintaining and updating the software and drivers is simpler.

These advantages have also attracted the robotics community, and, nowadays, robots are being prepared to connect to cloud computing infrastructures. This approach is called cloud robotics, which is a particular case of cloud computing that deals only with robots [18,19]. In Reference [20], the extensive resources of a cloud infrastructure are studied with a survey of cloud robotics and automation based on four of its features, namely, big data, cloud computing, collective robot learning and human computation. Cloud robotics allows for allocating resources in the cloud dynamically to support task offloading and information sharing in robotic applications [21]. Moreover, cloud robotics are expected to enhance robots capabilities such as speech recognition or mapping [22]. It can provide benefits in different applications, e.g., SLAM, navigation, voice recognition, weather monitoring, intrusion detection, surveillance, language translation, and communication. In the area of human–robot interaction, cloud robotics have substantially improved robots’ capabilities. The problem of personal robots adapting to changing environments, where people can provide information about them, is treated in [23]. In Reference [24], the cloud robotics approach is used to provide a robot with abilities for human–robot interaction and environmental sensing since it uses text-to-speech and speech recognition services.

Furthermore, there are also some applications in the area of image recognition, as in [25,26,27], where a cloud robotics system makes a robot recognize objects, even in the presence of uncertainties. In this way, cloud robotics enables robots to use remote servers to perform the heavier processing so that robots become smaller, smarter and cheaper [25]. In addition, the mobility of robots provides a means to collect ubiquitous data at homes, which enables exciting possibilities such as the processing of cloud-based data for human-aware homes. It is also possible to use the information available on the Internet and use it as a knowledge resource for autonomous service robots [26].

However, the use of cloud computing in this context has some important drawbacks that should be considered as well [25]:

- Robots need a permanent connection to keep full capabilities.
- It is necessary to protect the network from hackers.
- It is recommended to use on-board processing for strict real-time tasks such as those related to motion control and path planning.

In this article, we use the cloud robotics approach for integrating the robot Rovio with the Z-Wave controller Vera, both of them real, inexpensive off-the-shelf systems. This case study provides a compelling proof of concept that presents many of the aforementioned pros and cons. On the one hand, our integration provides users with improved capabilities for both the smart home system and the robot, e.g., image and voice recognition, which are way beyond the capabilities of these devices. On the other hand, the experiments that we have carried out in real homes show that the response time of the robot can become a problem for these types of applications.

The rest of the paper is organized as follows. In Section 2, the material and methods used are described: basic concepts of cloud computing and cloud robotics are introduced, and the integration of the surveillance robot Rovio in a Z-Wave network is described. Section 3 presents the cloud computing implementation developed and shows experimental results. The last section provides conclusions and future work.

## 2. Materials and Methods

In this section, we introduce briefly the main elements that compose our case study—a surveillance robot Rovio and the home automation controller for Z-Wave Vera —and how they are integrated into the same network.

### 2.1. Rovio

Rovio was released in 2008 and is a surveillance robot that has WiFi connectivity and provides an HTTP interface to control its capabilities. It has a set of sensors that includes a head-mounted moveable VGA camera, a microphone, infrared sensors for positioning and collision detection and a battery charge monitor. In addition, Rovio has the following actuators: a position controller for the robot neck, a speaker for two-way audio, three omni-directional wheels, and LED lights for illumination. There exists an API based on the HTTP protocol over TCP/IP that allows users to send commands to Rovio and to receive data from it. In particular, Rovio works using common gateway interface (CGI) commands within HTTP GET requests. For example, to retrieve the status report from Rovio, it is necessary to send the following command:

    http://ip_address/rev.cgi?Cmd=nav&action=1,
		
where ip_address is the IP address of Rovio in the network.

Finally, we must remark on one of the main features of Rovio: it can navigate autonomously through the home using infrared lights that are projected on the ceiling from its base. In order to extend the number of rooms where Rovio can navigate, devices known as *Rovio Truetrack Room Beacon* can be used. Each beacon must be set to a unique ID (1–9) using a selector to identify each room. Note that the production of Rovio has been discontinued, although there are other robots with similar and higher capabilities available in the market.

### 2.2. Vera

Vera is a controller for Z-wave, which is a wireless home automation technology based on IEEE 802.15.4. The role played by Vera is to act as a centralized controller in the network that offers the following services:

- It provides a platform for the creation of macros and scenarios in the home automation network.
- It works as a bridge between the Z-wave and Wifi, allowing the user to command his home automation devices via Wifi.
- It works as any router: it provides access to the local home network and to the Internet. In this way, a user can control the smart home network from the Internet. In addition, this provides a means to connect to an external cloud computing service.
- It provides a native UPnP interface, which allows integrating UPnP compliant devices in the smart home network.

### 2.3. Creation of a Basic Rovio Interface for Vera

One of the contributions of this paper is the integration of a service robot into a Z-wave network governed by Vera. An earlier version of our Vera’s Rovio adapter can be freely downloaded from [28]. Our first goal is to control the robot from Vera so it can be used as any other Z-wave device. In this way, the robot can be integrated into scenes or events that can be triggered from Vera depending on user actions, scheduling, or sensor information. For example, Rovio could be programmed to behave such as a mobile sensor that goes to a certain location to get a picture as a response to an event in the smart home, e.g., a change in the state of a sensor.

Vera uses an UPnP internal model for each device in the smart home network, even if the device is not UPnP compliant. Hence, if the device does not have an UPnP interface, it is necessary to create one. To this end, three different types of files have to be created There is an optional fourth type of file to provide a graphical user interface for the device in Vera. In this case, JavaScript Object Notation (JSON) is used to describe the appearance of the interface and how it interacts with the UPnP actions and variables defined in the other files. The details of this fourth file are omitted here since they are irrelevant for the scope of the paper: (1) a device description file; (2) service description files; and (3) implementation files. The first two types of files are UPnP compliant description files while the third one contains the implementation of the services in LUUP (Lua UPnP) or in JavaScript, which are the programming languages used by Vera.

For the Rovio plugin, the files uploaded are:

- The device description file that contains the top level information about Rovio’s plugin, and it is linked to the service files.
- The services’ description files, which list the services (actions and variables) that the device will provide.
- The implementation files that execute the actions to provide the services.
- An interface file in JSON format that allows the user to control the device using a graphical interface.

Once these files have been created and uploaded to Vera, Rovio is integrated into the Z-wave network.

**Figure 2:**
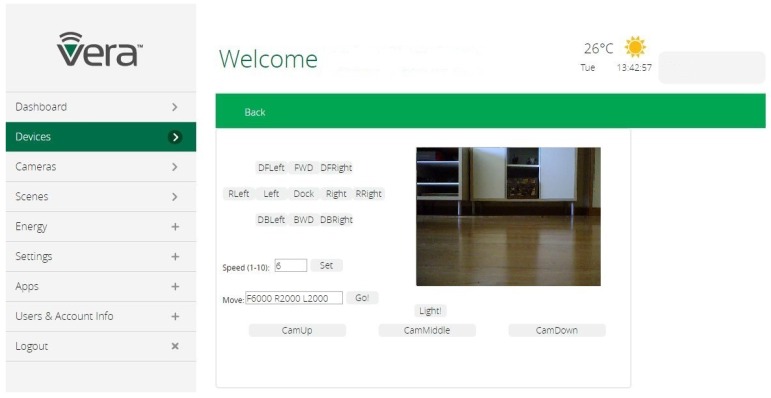
Vera Control Graphical Interface for Rovio.

In Figure 2, it can be seen the graphical user interface offered by Vera. On the top left of the interface, there are some buttons available to invoke the following Rovio movements: forward, backward, left, right (and their respective diagonals), the clockwise and counterclockwise rotation; and a button to send Rovio to its dock station. There is also a speed field available that users can modify to change Rovio’s velocity when it is controlled by using the buttons as mentioned earlier. On the top right of the interface, the image captured by Rovio’s webcam is shown. In addition, there is a button that turns on and off the LEDs installed on Rovio for low light environments. Another interesting functionality is the possibility to adjust Rovio’s cam position by means of three buttons (one for each position) on the interface. Finally, there is a field where a string that represents a sequence of movements can be inserted. This string contains several elements, where each element consists of a letter that indicates the movement direction and a number that represents the movement duration (in milliseconds). The movement directions implemented are: F (forward), B (backward), R (right), L (left), Z (counterclockwise rotation), and X (clockwise rotation). For example, if the string is F5000 R2000 B1600 L2000, Rovio goes forward for 5 s, it turns right for 2 s, then it goes backward for 1.6 s, and, finally, it turns left for 2 s. It must be noticed that the use of this string containing a sequence of movements for Rovio is another enhancement of its basic capabilities that could be implemented due to its integration with the router Vera. Given the simple nature of this new service, it can be run directly on Vera.

**Figure 3:**
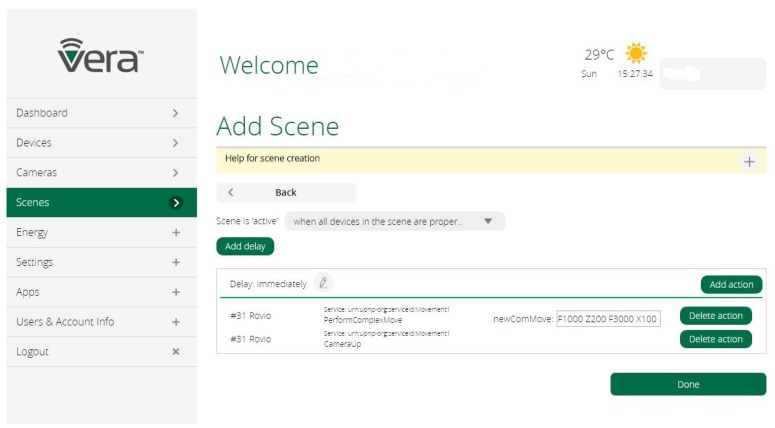
Scene section of Vera for the Rovio device.

The actions provided by Rovio are also available for macros and scenes, as shown in Figure 3. In this figure, it is possible to see how to program a movement string using the *PerformComplexMove* function implemented in the plugin. Likewise, these actions can be seen and invoked from any UPnP control point in the smart home network due to Vera’s native UPnP functionality.

The interaction with the device can also be accomplished by simple HTTP GET requests to Vera’s IP address with the following format: http://ip_address:3480/data_request?id=device&action=forward&device=31.

## 3. Results

Once the integration of Rovio and Vera is attained, we can focus on the development of advanced capabilities based on voice and image recognition. Given that Vera’s computation power is too low for these tasks, the oldest model, Vera 1, had a 240 MHz CPU and a 32 MB DDR RAM. The most advanced unit is Vera Plus, with an 880 MHz CPU and 256 MB DDR3 RAM. Cloud computing is used to overcome this issue. The entities that take part in the implemented cloud-based services are listed below:

- **User:** It is the actor that launches the Rovio service and sets the credentials through a GUI browser. It also interacts with Rovio by voice commands.
- **Vera Router:** It receives HTTP requests to execute actions and sends HTTP requests to Rovio’s web service by using the plugin developed.
- **GUI Browser:** It enables the user to interact with Vera by sending HTTP requests.
- **Rovio Web Service:** It receives requests from a Vera router located at a specific IP address and executes a Rovio application to manage them.
- **Rovio Application:** This is the main entity in the cloud. It receives the requests originated by Vera, performs computationally heavy tasks and sends the corresponding commands to Rovio, possibly by using Vera as a bridge.
- **Google Cloud Speech API:** It is an external cloud service that enables the Rovio application to convert audio into text.
- **Rovio Robot:** The robot receives the orders from the application in the cloud and performs tasks such as moving to a certain position or streaming audio.

**Figure 4:**
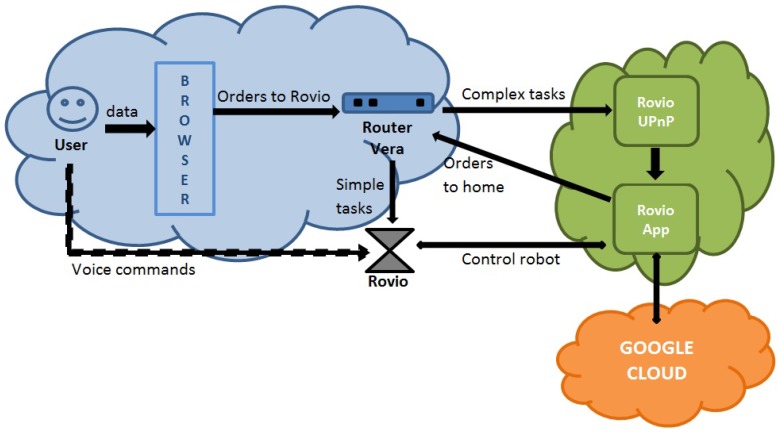
Entities’ interaction.

Figure 4 shows a block diagram that details the interaction between all the entities that work together.

### 3.1. Rovio, Home, and Cloud Interaction

A plugin for Vera has been developed to interoperate with the cloud. The plugin has the capability of requesting services by sending a label that identifies the operation and Rovio’s IP address. In this way, the remote cloud computing server has a means to exchange information with both Rovio and Vera.

The scenario used in our experiments consists of a Rovio and a Vera with Rovio’s plugin, which are available locally in the home. On the other side, a cloud server has been deployed with a web service that accepts HTTP requests. The Rovio plugin for Vera provides an interface to send HTTP requests to the web service in the cloud. The services implemented in Vera, the Rovio web service, and the Rovio application, which have also been tested in our experiments, are the following:

- Login Service: it allows the user to get access to the rest of the services. To invoke it, Vera must have the cloud server IP address and the web service URL to send the request, the service username, and password. An HTTP request is sent from Vera to the cloud service to log in to the web service system. This service has been implemented to authenticate the users in the platform, and it allows for invoking other services without refusing the requests.
- Rovio Connect Service: it allows the connection with Rovio. It requires Rovio’s IP address, username and password, which should be previously set in Rovio’s plugin as variables for Vera. Once this service is invoked, a Rovio application is launched in the cloud server with the information provided and the connection is established. The web service keeps a table with pairs of IP addresses from clients and Rovio application ports to manage different clients and future requests. The service has been implemented to maintain the connection between the cloud and Rovio. This service is essential as it provides connectivity with the robot.
- Launch Voice Recognition Service: it activates the voice recognition feature. Once this service is invoked, the Rovio robot receives a request, and it starts to stream audio data to the Rovio application.
- Stop Voice Recognition Service: it deactivates the voice recognition feature. This service stops the audio streaming from Rovio.

In addition to these services, many others can be implemented. For example, a *Garbage Detection Service* [7], which has not been used in our experiments, is available in Vera’s plugin, in the Rovio web service and also in the Rovio application. The router Vera provides the interface, the web service collects the request and then resends it to the Rovio application, which performs the task. This service allows for enabling/disabling a garbage detection algorithm. Another service that is used in our experiments and is only available in the Rovio application is the *Object Recognition Service*, which is invoked as a voice command.

Once all the elements are available, the user should send a login request to the Rovio Web Service in the cloud through the Vera interface. When the Rovio Web Service receives a login request, it launches a Rovio application to communicate with each client using a specific port. Then, a connection request should be sent to connect the cloud application with the robot. The Rovio Web Service identifies the client that sent the request and sends a request to the Rovio application using the corresponding port to connect to the robot. Finally, to process command voices, a request to activate the voice recognition service should be sent to the cloud server. Vera sends a request to Rovio’s Web Service, and it requests the Rovio application to collect audio streaming from the robot. In this way, the remote Rovio cloud application collects voice data from home and processes it by using the Google Cloud Speech API (Mountain View, CA, United States), which requires sending requests to an external cloud service. Once a voice command is analyzed and recognized, the cloud application sends the corresponding command to Rovio or Vera to execute the action according to the command voice.

The operation sequence is detailed below:

- The user logs in to Vera and accesses Rovio.
- The user sends the login HTTP request to the Web Service in the cloud through Rovio’s plugin in Vera. It authenticates the user in the cloud to get access to the rest of the services.
- The user sends the HTTP request to connect to the Web Service with Rovio’s IP address, the username, and the password. The service launches the cloud application locally with an associated port and stores the pair port–Vera IP address to identify a home with its corresponding application. Then, the service sends a request to the Rovio application with the provided parameters to establish a connection between Rovio’s application in the cloud and the robot at home.
- The user can send the voice activation HTTP request, and the service acts as a bridge and resends the request to the Rovio application in the cloud, which starts to collect audio data streams from Rovio’s microphone. The application records the audio periodically for two seconds and processes it by using the Google Cloud Speech API, which converts audio into text by applying powerful neural networks. The text retrieved from the audio is analyzed to check if it matches the commands loaded (four basic commands identify the four operations loaded at the beginning). When one of these operations is recognized, a new grammar is loaded to specify the information for the specific operation. The voice commands are configurable and included in an XML file whose fields and meanings are described below:
  – Move: when the voice command identified by this label is recognized, the Rovio application loads a new grammar that is composed of the paths saved in the robot and the two default routes, dock base and home, which are identified by *Place* labels inside the GoTo section. The user says the voice command defining the *Position* label and then the command that sets the path. Rovio, using its tracking beacons, will move to the position defined by the path.
  – Detect: once this voice command is recognized, the Rovio application loads the new grammar with the name of the objects identified by the *Object* labels inside the *ObjectDetection* section. The name of the object should be previously defined in the application, and a JPG image should be uploaded to look for the object. This application uses a SURF algorithm [27,29].
  – Action: when the Rovio application recognizes such voice command, it loads a grammar with the scenes defined in Vera. To this end, the Rovio application sends a request to Vera to retrieve the scenes, and then the application waits for the voice command that identifies the scene. When the application detects this command, it sends a request to Vera to execute the scene.
  – Cancel: when the application recognizes any voice command, it also adds the *Cancel* option to the grammar loaded. Then, when this voice command is recognized, the initial grammar is reloaded.

**Figure 5:**
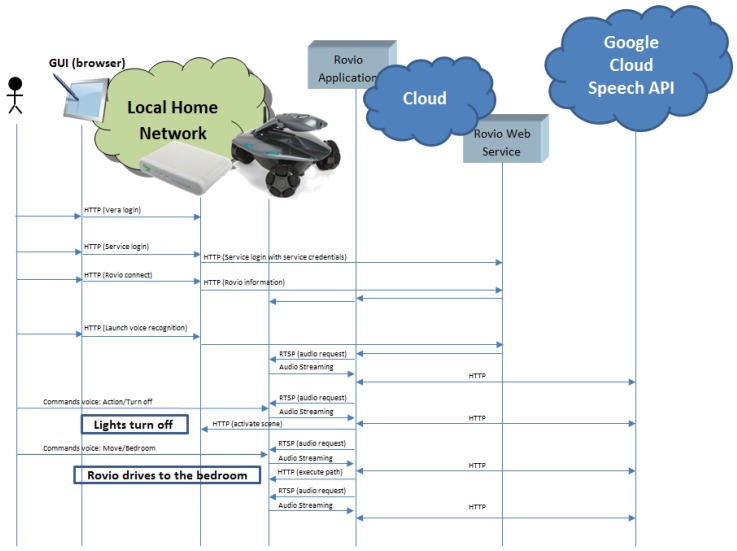
Messages exchange for Rovio and Vera router integration.

The working sequence described above is shown in Figure 5. In this diagram, the messages exchanged by the equipment at home, the Rovio application in the cloud, and the Google cloud are illustrated.

### 3.2. User Interaction

Regarding the user, he or she interacts with Rovio transparently using the local plugin installed in Vera. We have implemented a voice recognition service that allows the use of voice commands with Rovio. The speech recognition service relies on a list of possible voice commands that are stored in the cloud server in an XML file. The orders we have considered are:

- Follow a default path or one of the recorded paths available in Rovio,
- Begin the search of an object using the image recognition capability developed,
- Execute one of the scenes previously defined in Vera to trigger events in the house,
- Cancel a certain action.

The voice recognition service can be activated through the plugin. Once the user activates it, the plugin on Vera sends the corresponding message to the cloud server so the remote application can retrieve the information provided by Rovio’s microphone. There are important applications that stem from this service:

- It is possible to use voice commands to trigger events in the house as long as the user has Rovio nearby. In this way, Rovio behaves as a mobile microphone that provides an additional interface to communicate with the smart home.
- If Rovio is in a room identified by a Truetrack beacon, the user can say an operation to be performed (turn on/off the light, pull down/up the blinds) and the cloud server will command Vera to carry out the action in the room. That is, it is possible to use a basic level of context awareness with this service.
- The cloud server application for Rovio retrieves the scenes defined in Vera by sending a request. Therefore, the user can say a scene’s name to execute it and the cloud server application commands Vera to perform the corresponding actions.
- Another example is the activation of the object search algorithm using voice recognition: the user says the corresponding voice command and the name of the object to be found, and then Rovio starts to look for the object.

### 3.3. Experiments

We have carried out some experiments to test the cloud robotics implementation in a real home. For simplicity, we have used a computer on the local network to act as the cloud server. The deployment would be similar to that of a remote computer because the communication is set up by using the IP address. Moreover, the deployment of the Web Service and the Rovio Cloud Applications could be distributed among a remote server farm to gain speed. In addition, there is another cloud element that takes part in the experiments, the Google Cloud Speech API, which receives requests to identify the voice commands and sends the results to the Rovio cloud application.

In the following subsections, we present three different experiments to illustrate to what extent new services can emerge from the use of robots as mobile sensors and actuators in the smart home. Note that the experiments have been performed in a Spanish home equipped with Z-wave. Hence, the videos recorded are in Spanish, although English subtitles have been inserted to clarify what is happening in each experiment.

The purpose of these tests is to show a real case study where an inexpensive home automation system and a Rovio interact by using cloud-based mechanisms. These experiments show how heavy algorithms, such as object detection and voice recognition, can be easily implemented in the cloud. As can be seen in the following experiments, which can be seen in [30], the smart home becomes smarter due to the integration of a robot with cloud enhanced capabilities.

#### 3.3.1. Teddy Bear Search Experiment

The first experiment consists of requesting the robot to look for a teddy bear. Using the Vera interface, the user sends a login request to Rovio Web Service, which launches a Rovio application. Then, a connection request is sent, and, finally, the voice recognition algorithm is initiated. When the user says the *detect* command (Spanish command: *detectar*), the Rovio application loads the objects’ grammar available, and, then, once the user says the *teddy* command (Spanish command: *peluche*), it guides the robot and launches the SURF algorithm to look for the object indicated.

#### 3.3.2. New Object for Search Service Experiment

**Figure 6:**
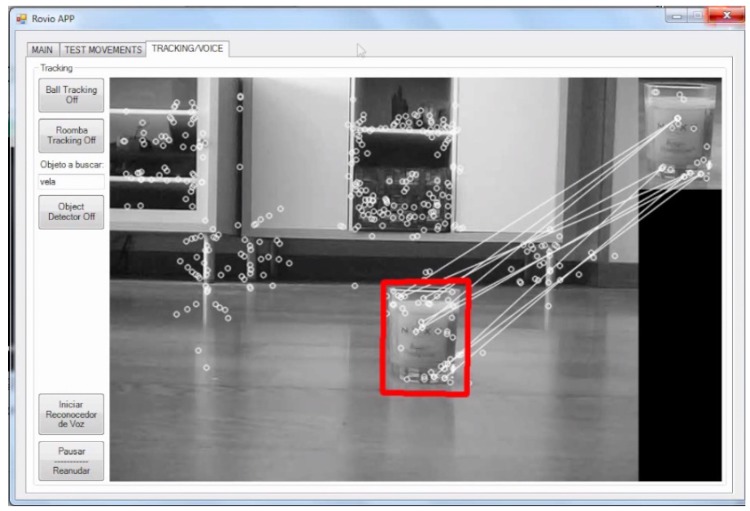
Rovio finds the candle object.

This experiment shows how easy is to insert a new object in the application. First, the user includes the object *candle* (*vela* in Spanish) in the XML file. Once the user says the command *candle*, the Rovio application ignores it and prints the message “The pattern image of the object to look for does not exist” (in Spanish: “La imagen patron del objeto a buscar no existe”), which indicates that the object pattern to compare with does not exist. To identify the object, the user takes a snapshot of the candle and saves it. Finally, the user tries again with the candle voice command, and, this time, the Rovio application launches the SURF algorithm and finds the object successfully, as it is shown in Figure 6.

#### 3.3.3. Z-Wave + Rovio Integration Experiment

Finally, in the last experiment, an integration scenario between Rovio and the smart home has been carried out. In this scenario, Rovio receives voice commands and performs the corresponding tasks by executing the scenes previously defined in Vera. When the Rovio application receives the *action* command (Spanish command: *accion*), it sends a request to Vera indicating the room where Rovio is located to perform the action. Firstly, the user says the *action* command (Spanish command: *accion*), and then the *turn off* command (Spanish command: *apagar*), and the Rovio application sends a request to Vera to turn off the lights in the room where Rovio is located (the bedroom). Next, the user orders Rovio to go to the living room by saying the voice commands *move* and *living room* (Spanish commands: *mover* and *salón*). Once the robot arrives at the living room, the user says the commands *action* and *turn on* (Spanish commands: *acción* and *encender*), and the Rovio application sends a request to Vera to turn on the lights in the room where Rovio is located (the living room). Finally, the user turns off the lights in the living room with the voice commands *action* and *turn off* (Spanish commands: *acción* and *apagar*), which send a request to Vera to perform the task. Note that the *turn off* command executes on Vera two different scenes depending on the room where the Rovio is located. When Rovio was in the bedroom, the *turn off* command triggered the scene for the bedroom, and, on the other hand, when Rovio was in the living room, the *turn off* command triggered the scene for the living room.

#### 3.3.4. Time Results

During the experiments, we have taken time measurements to compare the time response in the same scenario in a local network and a real cloud environment. In particular, we timed when the HTTP request is sent and the response is received. In the case of the audio streaming, we measured when we started to record the 3 s audio from Rovio and when it finished. All the measurements are displayed in Rovio’s console in real time and they were collected from several continuous executions.

The HTTP requests from Rovio application to Rovio and also the HTTP requests from Vera to Rovio Web Service have a latency in the order of milliseconds. The HTTP messages from Rovio application to Vera differ in tens of milliseconds. The audio streaming from Rovio to the Rovio application typically differ less than half a second. These are median values, though. However, in a certain streaming exchange, the delays become more than two seconds, which affects the real-time operation, and, in some cases, the robot does not interoperate in time. In Table 1, the time responses between the entities are detailed.

## 4. Conclusions

This paper shows a practical case study of how new advanced services can emerge in the smart home by using cloud computing to overcome the limitations of local computational resources. In particular, a Rovio robot has allowed us to offer services such as object detection and voice recognition. This is a remarkable achievement because it allows using a low-cost, off-the-shelf robot to provide sensing and actuation services that are only possible with much more sophisticated and expensive robots, e.g., Zenbo. Taking into account that we have developed an interface to integrate the service robot Rovio in a Z-wave network, which is also an inexpensive home automation technology, it can be concluded that users can enjoy the benefits of advanced cloud robotics at home inexpensively. For example, the overall cost of the hardware used in this work is below $500 and yet it allows for controlling the house using the voice and asking a robot to find an object for you.

Even when the experimental setup that we present in this paper is perfectly functional, we see it more as a platform for technical demonstration rather than a ready to market development. In particular, with the materials used, the delay is probably too large for real-world smart homes. A more realistic and practical setup would require much more computation power on the cloud side, which is feasible with the current technology and will be easier and cheaper as technology evolves. Our work also brings an important caveat in this regard: behind the cloud, there is a chain of software and hardware systems that are coordinated to provide a service. The quality of service provided by the cloud relies heavily on the adequate response of all the echelons that compose the chain. Given the tight time constraints of real-time operation, there is a need for services with deterministic time responses. Otherwise, the cause–effect relationship may not be perceived by the user, who may become frustrated with the robot and the smart home system. The success of this technology requires minimizing latency issues as long as there is a lack of technologies with deterministic time responses.

We believe that cloud robotics have a great potential to enhance the services provided by robots in the smart home. We also believe that there are strong economic incentives for the development of these services. On the one hand, it allows for simplifying hardware requirements so that the production costs and retail prices of both robots and smart home systems can be reduced. Likewise, cloud services are easier to update and maintain. Finally, it also has to be taken into account that the novel services that emerge from the integration of heterogeneous devices also increment the value that users perceive from their smart homes and service robots, which is one of the drivers of the technology industry.

Future work offers many different alternatives to expand our case study, which is simple at this stage and admits further development, e.g., to replace Google’s API for voice recognition or the algorithm used for object detection. In the first place, it is interesting to explore how to integrate and exploit the information from the home’s sensor network with our cloud system. Moreover, a scenario where multiple robots cooperate with the smart home system has enormous potential for the development of new services. In addition, robots become mobile sensors and actuators that can be exploited for data collection or actuation to improve people’s lives. Our approach allows, for example, sending the robot to the entrance door to take a picture if the door sensor is triggered. A Rovio equipped with a temperature sensor could be used for example to make a heat map of the home. Hence, new services have to be explored to exploit the possibilities that technology offers. From a technical viewpoint, the following step could be to integrate everything in the cloud to make devices at home as economical and simple as possible. This way, a central orchestrator entity would manage the communications among the cloud and the end devices at home, i.e., the smart home would have a single entry point to and from the cloud to orchestrate all the communications. The orchestrator could send messages to the cloud requesting services for different elements in the smart home and the responses would be resent to the corresponding smart home units that would have cloud enhanced capabilities. Therefore, the physical sensing and actuation would take place locally, but the processing of the information—the intelligence—would be in the cloud to save costs and to simplify upgrading and maintenance. 

## Figures and Tables

**Table 1:** Time response (in seconds).

	Local Network	Cloud Network
HTTP request from Rovio application to Rovio robot	0.0002–0.0012	0.0015–0.0017
3 s audio streaming from Rovio robot to Rovio application	3.19–5	3.49–7.28
HTTP request from Rovio application to router Vera	0.02–0.04	0.04–0.06
